# Self-rated health and the risk of incident type 2 diabetes mellitus: A cohort study

**DOI:** 10.1038/s41598-019-40090-y

**Published:** 2019-03-06

**Authors:** Jin-Won Noh, Yoosoo Chang, Minsun Park, Young Dae Kwon, Seungho Ryu

**Affiliations:** 1 Department of Healthcare Management and Institute of Global Healthcare Research, Eulji University, Seongnam, Republic of Korea; 2 Global Health Unit, Department of Health Sciences, University Medical Centre Groningen, University of Groningen, Groningen, The Netherlands; 3 Department of Occupational and Environmental Medicine, Kangbuk Samsung Hospital, Sungkyunkwan University School of Medicine, Seoul, Republic of Korea; 4 Center for Cohort Studies, Total Healthcare Center, Kangbuk Samsung Hospital, Sungkyunkwan University School of Medicine, Seoul, Republic of Korea; 5 Department of Clinical Research Design & Evaluation, SAIHST, Sungkyunkwan University, Seoul, Republic of Korea; 6 Department of Biobehavioral Health Science, College of Nursing, University of Illinois at Chicago, Chicago, IL USA; 7 Department of Humanities and Social Medicine, College of Medicine and Catholic Institute for Healthcare Management, The Catholic University of Korea, Seoul, Republic of Korea

## Abstract

We aimed to evaluate the association between self-rated health (SRH) and the risk of incident type 2 diabetes mellitus (T2D). This cohort study consisted of 250,805 Korean men and women without T2D at baseline. SRH was assessed at baseline with a self-administered structured questionnaire. Incident T2D was defined as fasting serum glucose ≥126 mg/dL, HbA1C ≥6.5%, or use of medication for T2D during follow-up. After adjustment for possible confounders including age, center, year of screening exam, smoking status, alcohol intake, physical activity, education level, total calorie intake, body mass index, sleep duration, depressive symptoms, family history of diabetes, history of hypertension, and history of cardiovascular disease, the multivariable-adjusted hazard ratios (95% confidence intervals) for incident T2D comparing good, fair, and poor or very poor SRH to very good SRH were 1.20 (0.98–1.48), 1.63 (1.33–1.98), and 1.83 (1.47–2.27), respectively. These associations were consistently observed in clinically relevant subgroups. Fair or poorer SRH was independently and positively associated with the development of T2D in a large-scale cohort study of apparently healthy Korean adults, indicating that SRH is a predictor of metabolic health. Physicians involved in diabetes screening and management should routinely consider SRH when evaluating T2D risk as well as overall health.

## Introduction

The prevalence and incidence of type 2 diabetes mellitus (T2D) is increasing worldwide, and is accompanied by considerable mortality^1^. In 2017, the International Diabetes Federation reported that 425 million people had diabetes worldwide, and this prevalence is expected to rise to 629 million by 2045^2^. In Korea and other Asian countries, diabetes has become a major cause of morbidity and mortality as well^3,4^. In total, 8.8% of Koreans had diabetes in 2017, and this prevalence is expected to rise to 12.1% by 2045^2^. T2D is associated with increased risk of cardiovascular, cancer, respiratory, and all-cause mortality and is related to poor quality of life^5,6^. Although the pathogenesis of T2D has not been fully elucidated, interrelated genetic, behavioral, and environmental risk factors are thought to contribute to the development of T2D^7^. Given the rising burden of T2D and its complications, it is important to develop preventive strategies for identifying high-risk individuals before they develop T2D and to identify potentially modifiable risk factors.

Self-rated health (SRH) is an individual’s subjective perception of their own health status and is one of the most widely used measures of general health in population health research^8^. SRH has attracted attention as an independent predictor of cardiovascular disease, stroke, lung disease, arthritis, functional impairment, depression, and overall mortality^9^. The strength of SRH is its ability to sum up the net effect of multiple risk factors, possibly including unmeasured risk factors^10^. SRH is associated with lifestyle factors such as physical activity, overweight or obesity, smoking, unhealthy meal planning choices, alcohol consumption, and genetic factors, all of which are also associated with T2D^11–13^. Prior studies have also reported a positive association between poorer SRH and elevated inflammatory markers, a risk factor for T2D^14–16^. Several studies found an association between poorer SRH and T2D using a cross-sectional design^17,18^, which is limited by the temporal ambiguity of exposure and outcome. Two cohort studies demonstrated that poorer SRH predicted increased risk of incident diabetes^19,20^. However, these studies did not consider important confounders such as sleep and depression^21,22^ while examining the association between SRH and a self-reported diagnosis of T2D, and they were performed in Western countries^17,19,20,23^. Studies on SRH have rarely included Asian groups, although cultural factors may affect SRH. Therefore, the aim of this cohort study was to evaluate self-rated health and the risk of incident T2D in a large sample of apparently healthy Korean men and women who participated in a health screening program.

## Materials and Methods

### Study population

The Kangbuk Samsung Health Study is a cohort study of Korean men and women who underwent a comprehensive annual or biennial health examination at the Kangbuk Samsung Hospital Health Screening Centers in Seoul and Suwon, South Korea^24^. Over 80% of participants were employees of various companies and local governmental organizations and their spouses. In South Korea, annual or biennial health screening exams of employees are required by the Industrial Safety and Health Law. The remaining participants voluntarily purchased a self-paid health checkup exam.

**Figure 1:**
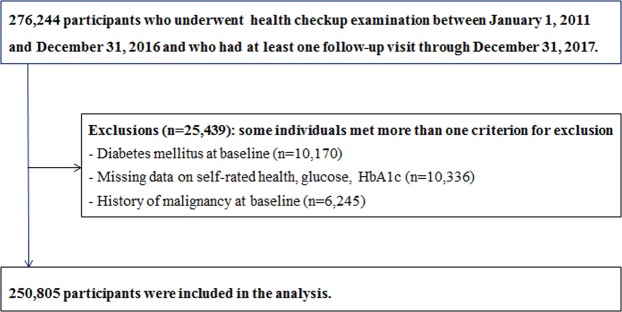
Selection of the study population. Of the people who were examined between January 1, 2011 and December 31, 2016, 276,244 had at least one follow-up visit by December 31, 2017, and 250,805 were eventually included in the study. T2D, type 2 diabetes mellitus.

The study population consisted of examinees who underwent comprehensive examination between January 1, 2011 and December 31, 2016 and who had at least one follow-up visit through December 31, 2017 (n = 276,244). We excluded participants who had any of the following conditions at baseline: missing data for SRH, glucose, or HbA1c (n = 10,336); a history of malignancy (n = 6,245); and T2D at baseline (n = 10,170). Because some individuals met more than one exclusion criterion, the total number of subjects included in the study was 250,805 (Fig. 1).

This study was designed to use de-identified data routinely collected as part of health screening examinations where questionnaire, blood tests and procedures (ultrasound, endoscopy, mammograms, etc.) are components of health screening exams. We use a computer system that automatically anonymizes and assigns study numbers for research purposes. The study was approved by the Institutional Review Board of Kangbuk Samsung Hospital (IRB No. KBSMC 2017-08-044), which waived the requirement for informed consent as we used only de-identified data obtained as part of routine health screening exams.

### Measurements

Data on demographic characteristics, health behaviors, education level, and medical history were collected by standardized, self-administered questionnaires, as previously described^25^. Health behaviors and education levels were categorized as follows: smoking status (never, former, or current smoker), alcohol consumption (≤20 g/day and >20 g/day), and education level (less than college graduate or college graduate or more). Family history of diabetes were defined as a self-reported diagnosis of diabetes in one or more first-degree relatives. Physical activity levels and sitting time were assessed using the validated Korean version of the International Physical Activity Questionnaire Short Form^25,26^. Physical activity levels were classified into three categories: inactive, minimally active, and health-enhancing physical activity (HEPA)^25^. Usual dietary consumption was assessed using a 106-item self-administered food frequency questionnaire designed and validated for use in Korea^27^. Sleep duration was assessed with the Pittsburgh Sleep Quality Index (PSQI)^28^. The PSQI has been validated for use in Korea^29^. One item in the PSQI asks about the duration of actual sleep at night in a typical 24 hour period over the past month. Sleep duration was categorized as ≤5, 6, 7, 8, or ≥9 hr. Depression was assessed using the Korean version^30^ of the Center for Epidemiologic Studies Depression (CES-D) Scale^31^. Depressive symptoms were defined as a CES-D score ≥16 and <25, and clinical depression was defined as a CES-D score ≥25. SRH was assessed at baseline using a self-administered questionnaire in Korean in order to measure general health^32^, defined by responses to a single question such as “In general, how would you rate your health?” with the possible choices being “very good” (1), “good” (2), “fair” (3), “poor” (4) or “very poor” (5)^33^. There have been a number of studies investigating the content validity of SRH in populations of various ethnicities and races^32,34^.

Height and weight were measured by trained nurses. Height was measured to the nearest 0.1 cm using a stadiometer with the examinee standing without shoes. Weight was measured in a light gown while barefoot to the nearest 0.1 kg using a bioimpedance analyzer (InBody 3.0 and Inbody 720, Biospace Co., Seoul Korea). Body mass index (BMI) was calculated as weight (kg) divided by height (m) squared (kg/m^2^) and obesity was defined as BMI ≥25 kg/m^2^, the proposed cutoff for diagnosis of obesity in Asian populations^35^. Blood pressure was measured using an automated oscillometric device (53000, Welch Allyn, New York, United States of America) by trained nurses while examinees were a sitting position with the arm supported at the heart level. Hypertension was defined as a systolic blood pressure ≥140 mmHg, diastolic blood pressure ≥90 mmHg, or current use of antihypertensive medication. T2D was defined as a fasting serum glucose ≥126 mg/dL, hemoglobin A1c (HbA1c) ≥ 6.5%, or current use of insulin or anti-diabetic medications.

Serum biochemical parameters were measured, including glucose, HbA1c, alanine aminotransferase (ALT), insulin, total cholesterol, triglycerides, low-density lipoprotein cholesterol (LDL-C), and high-density lipoprotein cholesterol (HDL-C) and are described in detail elsewhere^25^. Insulin resistance was assessed with the homeostatic model assessment – insulin resistance (HOMA-IR) equation: fasting blood insulin (uU/ml) × fasting blood glucose (mmol/l)/22.5. The Laboratory Medicine Department at Kangbuk Samsung Hospital in Seoul, Korea is accredited by the Korean Society of Laboratory Medicine and the Korean Association of Quality Assurance for Clinical Laboratories. The laboratory participates in College of American Pathologists Survey Proficiency Testing.

### Statistical analyses

Characteristics of the study participants were explored according to SRH and were compared according to SRH category using a linear regression test for continuous variables or a χ^2^ test for categorical variables. To test for linear trends, category numbers were used as continuous variables in regression models. SRH was categorized as follows; very good, good, fair, poor, or very poor. Since few subjects identified themselves as having very poor health (only 0.2%), we combined the poor and very poor categories.

Development of T2D was the primary endpoint of this study. Person-years were calculated as the sum of the follow-up duration from baseline until the development of T2D or until the final examination conducted prior to December 31 2017, whichever came first. The incidence rate was calculated as the number of incident cases divided by person-years of follow-up. Since we knew that T2D had occurred at some point between the two visits but did not know the precise timing of development, we used a parametric proportional hazard model to account for this type of interval censoring (*stpm* command in Stata)^36^. In these models, the baseline hazard function was parameterized with restricted cubic splines in log time with four degrees of freedom.

We estimated the adjusted hazard ratio (aHR) with a 95% confidence interval (CI) for incident T2D. Statistical models were initially adjusted for age and sex and then for year of screening exam, center, smoking status, alcohol intake, physical activity, family history of diabetes, education level, sleep duration, CES-D, total calorie intake, BMI, history of hypertension, and history of cardiovascular disease. Sensitivity analysis was conducted after implementing a 2-year wash-out period, during which any incident T2D cases identified were excluded. This approach was taken to assess if the original results may have been influenced by reverse causality in which T2D was present, but undiagnosed. We assessed the proportional hazards assumption by examining graphs of estimated log (-log) survival.

In addition, we performed stratified analyses in pre-specified subgroups defined by age (<50 vs. ≥50 years), sex (men vs. women), smoking (current smoker vs. noncurrent smoker), alcohol intake (<20 vs. ≥20 g/day), physical activity (no HEPA vs. HEPA), BMI (<25 vs. ≥25 kg/m^2^), HOMA-IR (<2.5 vs. ≥2.5), and high sensitivity C-reactive protein (hsCRP) (<1.0 vs. ≥1.0 mg/l). Interactions by subgroup characteristics were tested using likelihood ratio tests comparing models with and without multiplicative interaction terms. Statistical analyses were performed using STATA version 15.0 (StataCorp LP, College Station, TX, USA). All *p*-values were two-tailed, and *p*-values < 0.05 were considered statistically significant.

## Results

The baseline characteristics of the study population are described in Table 1. The mean (SD) age and BMI of study participants were 38.3 (7.7) years and 23.2 (3.3) kg/m^2^, respectively. Subjects with better SRH were more likely to be male, older, and highly educated and less likely to be obese, a current smoker, drink alcohol, or have a history of cardiovascular disease or hypertension. They also had lower levels of BMI (kg/m^2^), systolic BP (mmHg), total cholesterol (mg/dl), LDL-C (mg/dl), triglycerides (mg/dl), ALT (U/l), HOMA-IR, and CES-D and higher levels of HEPA (%), HDL-C (mg/dl), sleep duration, and total energy intake (kcal/day).

**Table 1:** Baseline characteristics of study participants by self-rated health.

Characteristics	Overall	Self-rated health category				*P* for trend
		Very good	Good	Fair	Poor or very poor	
Number	250,805	7,641	75,911	148,037	19,216	<0.001
Age (years)^a^	38.3 (7.7)	39.2 (8.8)	38.5 (8.0)	38.3 (7.5)	37.6 (7.7)	<0.001
Male (%)	55.0	63.8	60.3	52.6	49.0	<0.001
Current smoker (%)	27.8	24.0	27.0	28.3	29.8	<0.001
Alcohol intake (%)^b^	23.0	22.7	23.5	22.6	24.6	0.963
HEPA (%)	15.7	31.8	20.7	13.0	10.4	<0.001
High education level (%)^c^	84.1	85.1	86.3	83.4	80.3	<0.001
History of CVD (%)	0.8	0.6	0.7	0.8	1.6	<0.001
Hypertension (%)	9.3	6.8	8.00	9.6	13.1	<0.001
**Family history of diabetes (%)**						
Sleep duration (hours)	6.0 (6.0–7.0)	7.0 (6.0–7.0)	7.0 (6.0–7.0)	6.0 (6.0–7.0)	6.0 (6.0–7.0)	<0.001
CESD –D ≥ 16 (%)	11.2	3.6	5.4	12.0	30.1	<0.001
Obesity (%)	26.7	25.0	25.1	26.8	32.6	<0.001
BMI (kg/m^2^)	23.2 (3.3)	23.2 (2.8)	23.1 (3.0)	23.1 (3.3)	23.5 (4.1)	<0.001
Systolic BP (mmHg)^a^	109.0 (13.0)	109.3 (12.4)	109.3 (12.7)	108.8 (13.1)	108.5 (13.4)	<0.001
Diastolic BP (mmHg)^a^	69.8 (9.9)	69.5 (9.4)	69.8 (9.7)	69.9 (10.0)	69.8 (10.2)	0.058
Glucose (mg/dl)^a^	93.5 (8.5)	93.3 (8.6)	93.4 (8.5)	93.5 (8.5)	93.4 (8.6)	0.254
Total cholesterol (mg/dl)^a^	193.6 (33.7)	191.8 (32.4)	192.8 (32.8)	193.9 (34.0)	194.3 (35.6)	<0.001
LDL-C (mg/dl)^a^	119.6 (31.6)	117.5 (30.3)	118.9 (30.8)	120.0 (31.9)	120.5 (33.3)	<0.001
HDL-C (mg/dl)^a^	58.8 (15.1)	60.5 (15.2)	59.4 (15.0)	58.6 (15.1)	57.6 (15.0)	<0.001
Triglycerides (mg/dl)^d^	89 (63–132)	83 (60–121)	87 (63–127)	90 (64–134)	93 (66–141)	<0.001
ALT (U/l)^d^	18 (13–27)	17 (13–24)	18 (13–26)	18 (12–27)	18 (12–30)	<0.001
HOMA-IR^d^	1.16 (0.77–1.71)	1.04 (0.69–1.52)	1.10 (0.73–1.60)	1.18 (0.78–1.75)	1.28 (0.83–1.96)	<0.001
hsCRP (mg/l)^d^	0.4 (0.2–0.9)	0.4 (0.2–0.7)	0.4 (0.2–0.8)	0.4 (0.2–0.9)	0.5 (0.3–1.1)	<0.001
Total energy intake (kcal/d)^d,e^	1561.8 (1208.6–1953.9)	1592.8 (1208.6–2012.3)	1579.9 (1230.2–1971.6)	1548.6 (1196.9–1938.0)	1582.0 (1204.9–1990.4)	<0.001

Data are presented as ^a^Means (standard deviation), ^d^Medians (interquartile range), or percentages.

Abbreviations: ALT, alanine aminotransferase; BMI, body mass index; BP, blood pressure; CVD, cardiovascular disease; HDL-C, high-density lipoprotein-cholesterol; hsCRP, high sensitivity C-reactive protein; HOMA-IR, homeostasis model assessment of insulin resistance.

^b^≥20 g of ethanol per day ^c^≥college graduate.

^e^Among 175,345 participants with plausible estimated energy intake levels (within three standard deviations from the log-transformed mean energy intake).

Table 2 shows the development of diabetes according to SRH category. During 943,011.5 person-years of follow-up, 6,237 participants developed T2D (incidence rate 6.6 per 1,000 person-years) over a median follow-up period of 3.9 years (interquartile range, 2.1–5.3 years). Study participants were followed annually or biennially and the median frequency (interquartile range) of follow-up visits was 3 (2–5). The poorer SRH category was significantly associated with an increased incidence of T2D (*P* for trend <0.001). In the age and sex-adjusted model, HRs (95% CI) for T2D comparing the “good”, “fair” or “poor or very poor” vs. the “very good” self-rated health category were 1.15 (0.96–1.36), 1.76 (1.48–2.08), and 2.70 (2.25–3.24), respectively. This association persisted after further adjusting for center, year of screening exam, smoking status, alcohol intake, physical activity, education level, family history of diabetes, total calorie intake, BMI, history of hypertension, history of cardiovascular disease, sleep duration, and CES-D. Corresponding multivariable-adjusted HRs (95% CI) for T2D comparing the “good,” “fair,” or “poor or very poor” vs. the “very good” self-rated health category were 1.20 (0.98–1.48), 1.63 (1.33–1.98), and 1.83 (1.47–2.27), respectively. In sensitivity analysis after excluding subjects who developed T2D within the first 2 years of follow-up, the associations of SRH with incident T2D were essentially unchanged (Appendix Table 2).

**Table 2:** Development of diabetes by self-rated health category.

Self-rated health category	Person-years	Incident case	Incidence density (per 1,000 person-years)	Age and sex-adjusted HR (95% CI)	Multivariate HR^a^ (95% CI)
Very good	28,003.4	139	5.0	1.00 (reference)	1.00 (reference)
Good	290,116.1	1,501	5.2	1.15 (0.96–1.36)	1.20 (0.98–1.48)
Fair	555,687.4	3,929	7.1	1.76 (1.48–2.08)	1.63 (1.33–1.98)
Poor or very poor	69,204.6	668	9.7	2.70 (2.25–3.24)	1.83 (1.47–2.27)
P for trend				<0.001	<0.001

^a^Estimated from parametric proportional hazard models. The multivariable model was adjusted for age, sex, center, year of screening exam, smoking status, alcohol intake, physical activity, education level, total calorie intake, BMI, sleep duration, CESD, family history of diabetes, history of hypertension, and history of cardiovascular disease.

Abbreviations: BMI, body mass index; CESD, Center for Epidemiologic Studies Depression; CI, confidence intervals; HR, hazard ratios.

In pre-specified subgroup analyses (Table 3), associations between SRH and incident diabetes were stronger patients with an alcohol intake of <20 g/day compared to those with an alcohol intake of ≥20 g/day (*P* for interaction = 0.009). Otherwise, their associations were similar with no significant interactions between subgroups stratified by age (<50 vs. ≥50 years), sex (women vs. men), smoking status (noncurrent smoker vs. current smoker), alcohol intake (<20 vs. ≥20 g/day), physical activity (no HEPA vs. HEPA), BMI (<25 vs. ≥25 kg/m^2^), HOMA-IR (<2.5 vs. ≥2.5) or hsCRP (<1.0 vs. ≥1.0 mg/L).

**Table 3:** Hazard ratios^a^ (95% CI) of incident diabetes according to self-rated health category in clinically relevant subgroups.

Subgroup	Self-rated health category				*P* for trend	*P* for interaction
	Very good	Good	Fair	Poor or very poor		
**Age**						0.327
<50 years (N = 231,069)	Reference	1.10 (0.87–1.38)	1.51 (1.20–1.89)	1.61 (1.27–2.06)	<0.001	
≥50 years (N = 19,736)	Reference	1.46 (0.96–2.25)	1.73 (1.14–2.64)	1.93 (1.19–3.12)	<0.001	
**Sex**						0.880
Female (N = 112,931)	Reference	1.01 (0.63–1.62)	1.42 (0.90–2.24)	1.59 (0.99–2.57)	<0.001	
Male (N = 137,874)	Reference	1.24 (0.99–1.55)	1.67 (1.34–2.09)	1.88 (1.47–2.39)	<0.001	
**Current smoking**						0.826
No (N = 163,449)	Reference	1.20 (0.92–1.58)	1.66 (1.27–2.17)	1.91 (1.43–2.55)	<0.001	
Yes (N = 63,062)	Reference	1.26 (0.87–1.81)	1.65 (1.16–2.36)	1.81 (1.24–2.63)	<0.001	
**Alcohol intake**						0.009
<20 g/day (N = 181,455)	Reference	1.35 (1.01–1.79)	1.88 (1.42–2.48)	2.37 (1.76–3.18)	<0.001	
≥20 g/day (N = 54,208)	Reference	1.05 (0.77–1.44)	1.33 (0.98–1.80)	1.34 (0.96–1.87)	<0.001	
**HEPA**						0.375
No (N = 209,494)	Reference	1.18 (0.91–1.52)	1.57 (1.23–2.02)	1.82 (1.40–2.37)	<0.001	
Yes (N = 39,068)	Reference	1.22 (0.87–1.72)	1.75 (1.26–2.45)	1.59 (1.05–2.41)	<0.001	
**Body mass index**						0.331
<25 kg/m^2^ (N = 183,745)	Reference	1.00 (0.75–1.32)	1.34 (1.02–1.76)	1.52 (1.11–2.07)	<0.001	
≥25 kg/m^2^ (N = 66,871)	Reference	1.43 (1.06–1.92)	1.94 (1.45–2.60)	2.20 (1.62–2.99)	<0.001	
**HOMA-IR**						0.822
<2.5 (N = 226,602)	Reference	1.10 (0.88–1.37)	1.45 (1.17–1.81)	1.58 (1.23–2.02)	<0.001	
≥2.5 (N = 22,710)	Reference	1.38 (0.84–2.28)	1.80 (1.10–2.95)	2.05 (1.23–3.39)	<0.001	
**hsCRP**						0.683
<1.0 mg/l (N = 160,098)	Reference	1.34 (0.99–1.80)	1.74 (1.29–2.34)	2.05 (1.49–2.83)	<0.001	
≥1.0 mg/l (N = 45,947)	Reference	1.32 (0.88–2.00)	1.84 (1.23–2.76)	1.96 (1.29–2.99)	<0.001	

^a^Estimated from parametric proportional hazard models adjusted for age, sex, center, year of screening exam, smoking status, alcohol intake, physical activity, education level, total calorie intake, BMI, sleep duration, CES-D, family history of diabetes, history of hypertension and history of cardiovascular disease.

Abbreviations: HEPA, health-enhancing physical activity; hsCRP, high sensitivity C-reactive protein; and HOMA-IR, homeostasis model assessment.

## Discussion

In a large-scale cohort study of 250,805 young and middle-aged Korean men and women, poorer SRH was significantly associated with a higher risk of T2D in a dose-dependent manner. In our study, poorer SRH showed a graded positive association with depressive symptoms, which is in line with previous studies^37,38^. However, the association of fair or poorer SRH with incident T2D persisted even after adjustment for potential confounders, including demographic characteristics, anthropometrics, comorbidities, lifestyle factors and depressive symptoms, indicating that the association of SRH with the development of T2D cannot be fully explained by health-related variables, depression, or other comorbidities. Our findings suggest that SRH may be an independent predictor of metabolic disease such as diabetes, even in a relatively healthy low-risk population.

Although SRH has been investigated in various health conditions such as cardiovascular disease, stroke, lung disease, arthritis, functional impairment, depression, and overall mortality^9^, only two previous cohort studies examined the association between SRH and T2D incidence in Europe and the United States. In a population-based prospective case-cohort study of 3,399 incident type 2 diabetic case participants and a subcohort of 4,619 participants across several European countries with a mean follow-up of 9.1 years, Wennberg *et al*. reported that low SRH (moderate or poor) was associated with a higher risk of T2D compared with high (excellent or good), with a multivariable-adjusted HR (95% CI) of 1.29 (1.09–1.53)^19^. In our study, an almost two-fold increase in risk for incident T2D was observed with poor or very poor SRH compared to the very good SRH category, with a fully-adjusted HR (95% CI) of 1.83 (1.47–2.27). Another cohort study by Latham and Peek evaluated the association between SRH and self-diagnosis of major chronic diseases including diabetes among 4,770 adults aged 51 to 61 years^20^. In this study, each unit increase in SRH (higher scores indicate better health) was inversely associated with a 0.82 times lower risk of subsequent T2D^20^. The different findings across studies could be attributable to differences in sample size, study population (age, ethnicity, and sex composition), measures of T2D, use of a reference group and controlling for potential confounding factors. In particular, no previous studies determined incident diabetes based on objective measures such as laboratory testing of fasting blood glucose and HbA1c. This is important, as some proportion of diabetic patients can remain undiagnosed without screening, resulting in misclassification errors when detecting T2D as an outcome^39^. Furthermore, SRH can be related to sleep and mental health issues such as depression, but none of the former studies considered those to be important confounders^21,40^. In our study, poorer SRH showed graded and positive association with depressive symptoms^37,38^. Likewise, in previous studies, depressive symptoms were significantly associated to with poorer SRH^37,38^. In our study, fair or poorer SRH was independently associated with higher risk of incident T2D even after adjustment for depressive symptoms and other confounders.

The strengths of the present study are the large sample size, the prospective cohort study design, the use of carefully standardized clinical and laboratory procedures, and the availability of data on mental health and lifestyle factors including smoking, alcohol consumption, physical activity, diet, and sleep. More importantly, in our study, fasting blood glucose, HbA1c, and other covariates were measured by annual or biennial laboratory and physical examinations; thus, T2D diagnoses were made based on objective measures, unlike previous cohort studies. The mean age of the participants in the former study by Wennberg *et al*. and by Latham and Peek was 48.8 years and 55.3 years, respectively^19,20^, while our study population was much younger, with a mean age of 38.3 years. Furthermore, the study population consisted of asymptomatic examinees who participated in the health screening examination program; thus, the study findings from the low-risk population are less likely to be affected by survivor bias and biases related to comorbidities and use of multiple medications, compared to findings from previous cohorts. The present study, which used objective measures to diagnose T2D, demonstrated that poor SRH was significantly associated with an increased risk of T2D development, even in low-risk young Korean adults.

In the present study, we observed that a positive association between SRH and new-onset T2D was stronger in patients with an alcohol intake of <20 g/day compared to those with a higher alcohol intake of ≥2 g/day. The reasons for this are unclear, but people tend to reduce alcohol intake due to their comorbidities or health concerns. Thus, these findings may be explained by the fact that the group who drank <20 g/day could have included former drinkers who might have reduced or stopped their alcohol intake because of other health issues^41^, indicating that poor SRH in those who drank <20 g/day might imply a worse unmeasured condition. In addition, in most studies, moderate drinkers have been found to have good SRH compared with other types of drinkers^42^, and moderate drinkers have reported better health than nondrinkers^43^. Due to the use of multiple comparisons, chance may be another explanation for the observed difference across subgroups.

The exact mechanisms by which SRH predicts new-onset DM are not fully understood. T2D is closely related to unhealthy lifestyle habits that result in obesity, insulin resistance, and dyslipidemia. When people are faced with the question, “In general, how do you rate your health?” they may look back on their overall lifestyle and habits to evaluate and rate their health, beyond what is explained by objective measures that many studies usually assess. Studies have shown that SRH is associated with lifestyle factors such as exercise, overweight or obesity, and smoking, unhealthy meal planning choices, alcohol consumption, and genetic factors, all of which also have been associated with T2D^11–13^. In addition, because SRH involves a subjective health assessment that is influenced by different dimensions such as sociodemographic, physical, and psychological factors^44,45^, it may well reflect an individual’s specific situation. In addition, studies showed that poor self-rated health was associated with elevated inflammatory markers, a predictor for T2D, in adolescents and older adults^14–16^. Future studies are required to elucidate the mechanism underlying the predictive role of SRH on T2D development.

A few study limitations should be acknowledged. First, T2D was diagnosed based on single measurements of fasting glucose and HbA1c, whereas diagnostic criteria recommend confirmation by repeated testing; however, HbA1c has good pre-analytical stability and is less likely to be affected by acute perturbations (e.g., stress, exercise, or smoking)^46^. Second, waist circumference measurements, however, were available only in a fraction of study participants, limiting our ability to adjust central obesity as a confounder, which is a stronger indicator for insulin resistance and T2D incidence than BMI, especially in Asian populations^44^. Third, the SRH question used in this study was not directly validated in our study population, but previous studies have reported that SRH has high predictive and concurrent validity, as measured by its association with subsequent mortality and various measures of health outcomes^8,9,47^. Studies have also reported that the reliability of SRH is reliable in both adolescents and adult populations^48–50^. Fourth, a relatively short follow-up period with a median follow-up of 3.9 years (interquartile 2.1–5.3 years, up to 6.9 years) is a limitation of this study. We performed sensitivity analysis by excluding subjects who developed T2D within the first 2 years of follow-up in order to consider whether the original results may have been influenced by reverse causality in which T2D was present, but undiagnosed. In this sensitivity analysis, the association between SRH and incident T2D was essentially unchanged (Appendix Table 2). Also, our study findings are in line with previous studies on the association between SRH and T2D that involved longer follow-up periods (9.1 years)^19^. Further studies with longer follow-up are required to determine the long-term predictive role of SRH on incident T2D. Fifth, information on SRH, medical history, lifestyle variables and depressive symptoms were collected using self-administered structured questionnaires. The possibility of measurement errors cannot be excluded for those variables, which might have resulted in some degree of residual confounding. Lastly, the study population consisted of young and middle-aged Koreans who regularly attended health-screening exams; thus, our findings might not be generalizable to other age groups, to populations with a higher prevalence of comorbidities, or to other racial/ethnic groups.

In conclusion, poor SRH was significantly associated with increased risk of developing T2D in young and middle-aged Korean men and women, after adjusting for confounding factors. Further mechanistic research is needed to elucidate the mechanism by which SRH predicts the incidence of T2D. Given the established value of SRH, one of the most widely used measures of general health in population health research, for predicting various chronic diseases including T2D, clinicians involved in diabetes screening and management should routinely consider SRH when evaluating T2D risk as well as overall health.

## Supplementary information

Supplementary tables
