# Supplementary material for: Self-rated health and the risk of incident type 2 diabetes mellitus: A cohort study
Source: Sci Rep. 2019 Mar 6;9:3697. doi: 10.1038/s41598-019-40090-y (PMC6403398; doi:10.1038/s41598-019-40090-y)
Supplement: Supplementary file 1 — Supplementary tables [file 41598_2019_40090_MOESM1_ESM.pdf]

Self-rated health and the risk of incident type 2 diabetes mellitus: A cohort study

Jin-Won Noh[1,2], Yoosoo Chang[3,4,5*], Minsun Park[6], Young Dae Kwon[7], Seungho Ryu[3,4,5*]

[1]*Department of Healthcare Management and Institute of Global Healthcare Research, Eulji University, Seongnam, Republic of Korea;* [2]*Global Health Unit, Department of Health Sciences, University Medical Centre Groningen, University of Groningen, Groningen, the Netherlands;* [3]*Department of Occupational and Environmental Medicine, Kangbuk Samsung Hospital, Sungkyunkwan University School of Medicine, Seoul;* [4]*Center for Cohort Studies, Total Healthcare Center, Kangbuk Samsung Hospital, Sungkyunkwan University School of Medicine;* [5]*Department of Clinical Research Design & Evaluation, SAIHST, Sungkyunkwan University, Seoul, South Korea;* [6]*Department of Biobehavioral Health Science, College of Nursing, University of Illinois at Chicago, Chicago, IL, USA;* [7]*Department of Humanities and Social Medicine, College of Medicine and Catholic Institute for Healthcare Management, The Catholic University of Korea, Seoul, Republic of Korea*

Running title: Self-rated health and type 2 diabetes

Address for correspondence: Seungho Ryu, MD, PhD, Kangbuk Samsung Hospital, Samsung Main Building B2, 250, Taepyung-ro 2ga, Jung-gu, Seoul, South Korea 04514

E-mail: sh703.yoo@gmail.com. Telephone: 82-2-2001-5137. Fax: 82-2-757-0436.

Co-corresponding author: Yoosoo Chang, MD, PhD, Kangbuk Samsung Hospital, Samsung Main Building B2, 250, Taepyung-ro 2ga, Jung-gu, Seoul, South Korea 04514

E-mail: yoosoo.chang@gmail.com. Telephone: 82-2-2001-5139. Fax: 82-2-757-0436.

Appendix Table 1. Cumulative incidence ratio (CIR) of incident diabetes by self-rated health category

**Self-rated health category**	Number	Incident case	Cumulative incidence rate (per 100 persons)	Age and sex-adjusted CIR (95% CI)	Multivariate CIR[a] (95% CI)
Very good	7,641	139	182.0	1.00 (reference)	1.00 (reference)
Good	75,911	1,501	198.0	1.19 (1.00-1.41)	1.21 (0.99-1.48)
Fair	148,037	3,929	265.0	1.77 (1.50-2.09)	1.64 (1.35-1.99)
Poor or very poor	19,216	668	348.0	2.52 (2.10-3.01)	1.82 (1.47-2.25)
P for trend				<0.001	<0.001

[a]Estimated from Poisson regression with robust error variance. The multivariable model was adjusted for age, center, year of screening exam, smoking status, alcohol intake, physical activity, education level, total calorie intake, BMI, sleep duration, CESD, family history of diabetes, history of hypertension, and history of cardiovascular disease.

Abbreviations: BMI, body mass index; CI, confidence intervals; HR, hazard ratios.

Appendix Table 2. Development of T2D by self-rated health category after excluding 2,236 cases occurring within two years.

**Self-rated health category**	Person-years	Incident case	Incidence density (per 1,000 person-years)	Age and sex-adjusted HR (95% CI)	Multivariate HR[a] (95% CI)
Very good	27,921.4	77	2.8	1.00 (reference)	1.00 (reference)
Good	289,431.2	970	3.4	1.29 (1.02-1.63)	1.35 (1.04-1.78)
Fair	553,766.7	2,526	4.6	1.98 (1.57-2.48)	1.78 (1.36-2.32)
Poor or very poor	6,8869.1	428	6.2	3.11 (2.44-3.97)	1.99 (1.49-2.65)
P for trend				<0.001	<0.001

[a]Estimated from parametric proportional hazard models. The multivariable model was adjusted for age, center, year of screening exam, smoking status, alcohol intake, physical activity, education level, total calorie intake, BMI, sleep duration, CESD, family history of diabetes, history of hypertension, and history of cardiovascular disease.

Abbreviations: BMI, body mass index; CI, confidence intervals; HR, hazard ratio.

Appendix Table 3. Development of T2D by self-rated health category using different categories as the reference

**Self-rated health category**	Multivariate HR[a] (95% CI)	P value [b]	Multivariate HR[a] (95% CI)	P value [c]	Multivariate HR[a] (95% CI)	P value [d]
Very good	1.00 (reference)		0.84 (0.68-1.02)	0.082	0.62 (0.50-0.75)	<0.001
Good	1.20 (0.98-1.48)	0.082	1.00 (reference)		0.74 (0.69-0.79)	<0.001
Fair	1.63 (1.33-1.98)	<0.001	1.36 (1.27-1.45)	<0.001	1.00 (reference)	
Poor or very poor	1.83 (1.47-2.27)	<0.001	1.52 (1.37-1.70)	<0.001	1.12 (1.02-1.23)	0.015

[a]Estimated from parametric proportional hazard models. The multivariable model was adjusted for age, center, year of screening exam, smoking status, alcohol intake, physical activity, education level, total calorie intake, BMI, sleep duration, CESD, family history of diabetes, history of hypertension, and history of cardiovascular disease.

[b]P value for comparing each SRH category to very good.

[c]P value for comparing each SRH category to good.

[d]P value for comparing each SRH category to fair.
